# *Shewanella* sp. O23S as a Driving Agent of a System Utilizing Dissimilatory Arsenate-Reducing Bacteria Responsible for Self-Cleaning of Water Contaminated with Arsenic

**DOI:** 10.3390/ijms160714409

**Published:** 2015-06-25

**Authors:** Lukasz Drewniak, Robert Stasiuk, Witold Uhrynowski, Aleksandra Sklodowska

**Affiliations:** Laboratory of Environmental Pollution Analysis, Faculty of Biology, University of Warsaw, Miecznikowa 1, 02-096 Warsaw, Poland; E-Mails: r.stasiuk@biol.uw.edu.pl (R.S.); w.uhrynowski@biol.uw.edu.pl (W.U.); asklodowska@biol.uw.edu.pl (A.S.)

**Keywords:** *Shewanella* spp., dissimilatory arsenate reduction, arsenic removal

## Abstract

The purpose of this study was a detailed characterization of *Shewanella* sp. O23S, a strain involved in arsenic transformation in ancient gold mine waters contaminated with arsenic and other heavy metals. Physiological analysis of *Shewanella* sp. O23S showed that it is a facultative anaerobe, capable of growth using arsenate, thiosulfate, nitrate, iron or manganite as a terminal electron acceptor, and lactate or citrate as an electron donor. The strain can grow under anaerobic conditions and utilize arsenate in the respiratory process in a broad range of temperatures (10–37 °C), pH (4–8), salinity (0%–2%), and the presence of heavy metals (Cd, Co, Cr, Cu, Mn, Mo, Se, V and Zn). Under reductive conditions this strain can simultaneously use arsenate and thiosulfate as electron acceptors and produce yellow arsenic (III) sulfide (As_2_S_3_) precipitate. Simulation of As-removal from water containing arsenate (2.5 mM) and thiosulfate (5 mM) showed 82.5% efficiency after 21 days of incubation at room temperature. Based on the obtained results, we have proposed a model of a microbially mediated system for self-cleaning of mine waters contaminated with arsenic, in which *Shewanella* sp. O23S is the main driving agent.

## 1. Introduction

Arsenic reduction may occur naturally in the environment as a result of the impact of physical and chemical factors or it can be caused by microorganisms capable of transforming arsenic compounds. Bacterial reduction of arsenate into arsenite may proceed by two different mechanisms. The first is related to the detoxification of the cells, and involves cytoplasmic reduction of arsenates into arsenites, which are subsequently removed from the cell by efflux pumps. In the second reduction process, called dissimilatory reduction, bacterial cells use arsenate as a terminal electron acceptor in the respiration process. Both reduction mechanisms constitute essential elements of the biogeochemical cycle of arsenic since they are responsible for the transformation of soluble As(V) compounds into more toxic and less mobile arsenites. Microorganisms carrying out cytoplasmic arsenate reduction are found in both aerobic and anaerobic environments as their respiration preferences are not connected to this detoxification process. In contrast, dissimilatory arsenate reduction is more prevalent in anoxic environments and most of the arsenate-respiring bacteria are obligatory anaerobes [1]. It has been presented in several studies that dissimilatory arsenate-reducing bacteria (DARB) may reduce and release arsenic adsorbed on the surface of iron minerals such as ferrihydrite, goethite, or hematite [2,3,4]. Interestingly, some of the bacteria capable of reducing As(V) to As(III) have been shown to be involved in the formation of diverse insoluble arsenic minerals [5,6], and thus may potentially contribute to the purification of the environment by arsenic immobilization. The role of microbial arsenic reduction in the homeostasis of this element in the environment depends on the physical and chemical conditions prevailing in the ecosystems, as well as on the structure of the microbial community involved in biogeochemical processes, such as dissolution and precipitation of (arsenic) minerals.

An interesting example of the environment for studying the role of DARB in the biogeochemical cycle of arsenic are effluent waters and bottom sediments in an ancient gold mine in Zloty Stok (south-western Poland), since (i) both inorganic arsenic forms are present; (ii) the coexistence of arsenite oxidizers and dissimilatory arsenate reducers in the microbial community was confirmed [7], and, most importantly; (iii) microbial mats that inhabit the bottom sediments in the mine form a natural barrier trapping arsenic and other heavy metals leaking from the dewatering system [8].

In our previous studies we have focused on DARB in the context of their contribution in arsenic mobilization and, consequently, its dissemination into the environment [9,10]. We have isolated two strains of DARB: *Shewanella* sp. O23S and *Aeromonas* sp. O23A, which are able to mobilize arsenic from mine rocks containing arsenic minerals [9]. It has been shown that under anaerobic conditions these strains are able to grow on minimal salt medium supplemented with sodium lactate and release arsenic from rocks containing arsenopyrite–FeAsS, loellingite–FeAs_2_, traces of scorodite–FeAsO_4_-2H_2_O, and origin ore. In turn, for several other arsenic-respiring bacteria isolated from the Zloty Stok mine, we have demonstrated the ability to mobilize arsenic from As-bearing copper minerals (containing enargite and tennantite) [10].

The objective of the present study was a detailed physiological analysis of *Shewanella* sp. O23S, the dominant strain within the DARB community found in Zloty Stok. The analyses were focused on the characterization of dissimilatory arsenate reduction and other associated processes in the context of their role in colonization and purification of arsenic-contaminated waters.

## 2. Results

### 2.1. Phylogeny of Shewanella sp. O23S

**Figure 1:**
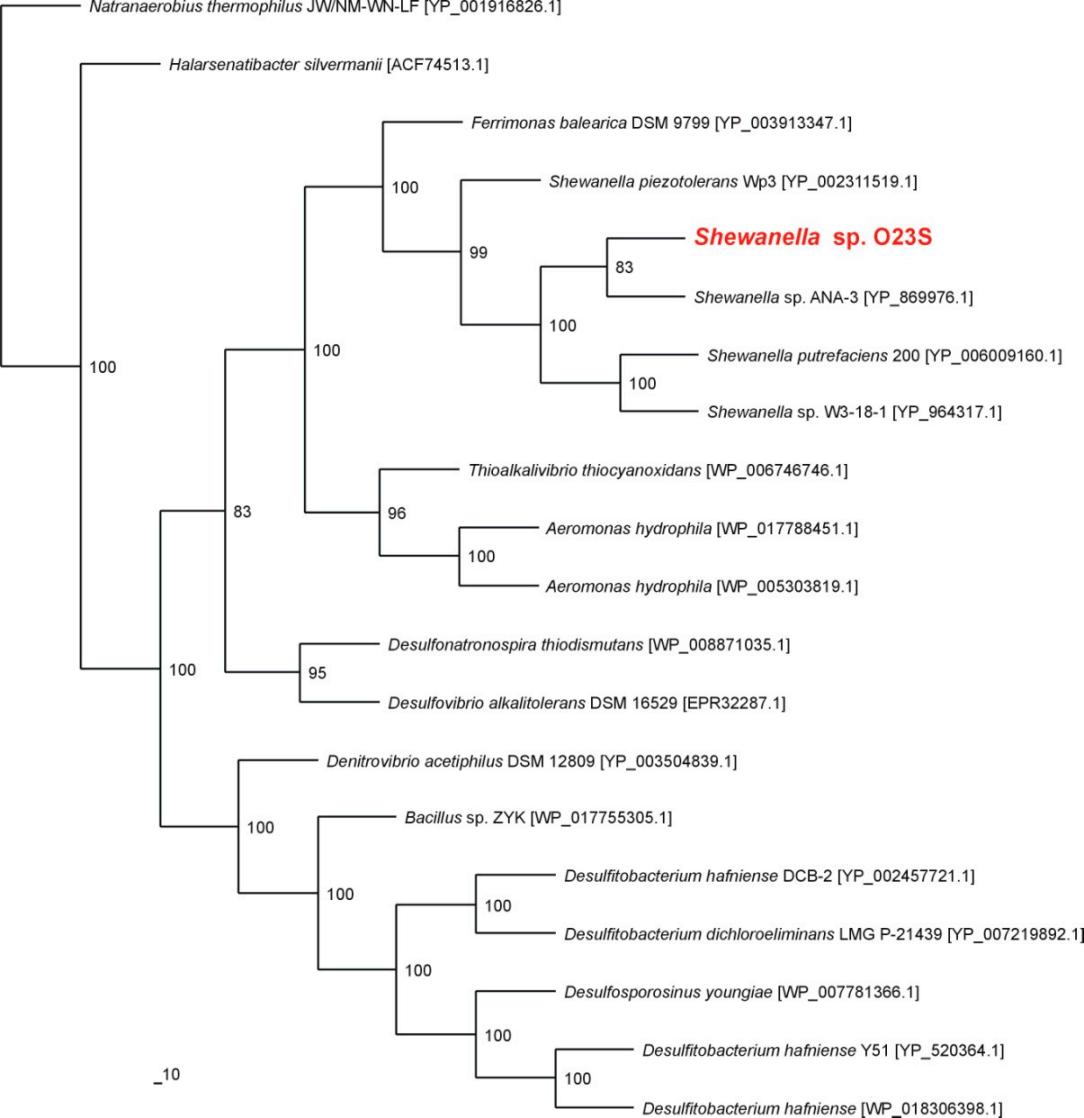
Phylogenetic tree of the dissimilatory arsenate reductase ArrA based on 20 sequences. The investigated strain is indicated in red. The unrooted tree was constructed using the Maximum Likelihood algorithm according to Jones-Taylor-Thornton (JTT) model. The statistical support for the internal nodes was determined by 100 bootstrap replicates. Accession numbers of the protein sequences used for the phylogenetic analysis are given in parentheses.

Taxonomic analysis of the 16S rRNA gene placed the strain O23S with other *Shewanella* spp. The most closely related sequences found in the GenBank belong to *Shewanella* sp. W3-18-1 and *Shewanella putrefaciens* (99% similarity for the 16S rRNA gene for both the strains). Phylogenetic analysis of the predicted dissimilatory arsenate reductase (ArrA) showed that the strain O23S is closely related with the well-characterized arsenate-respiring strain *Shewanella* sp. ANA-3 (Figure 1).

**Figure 2:**
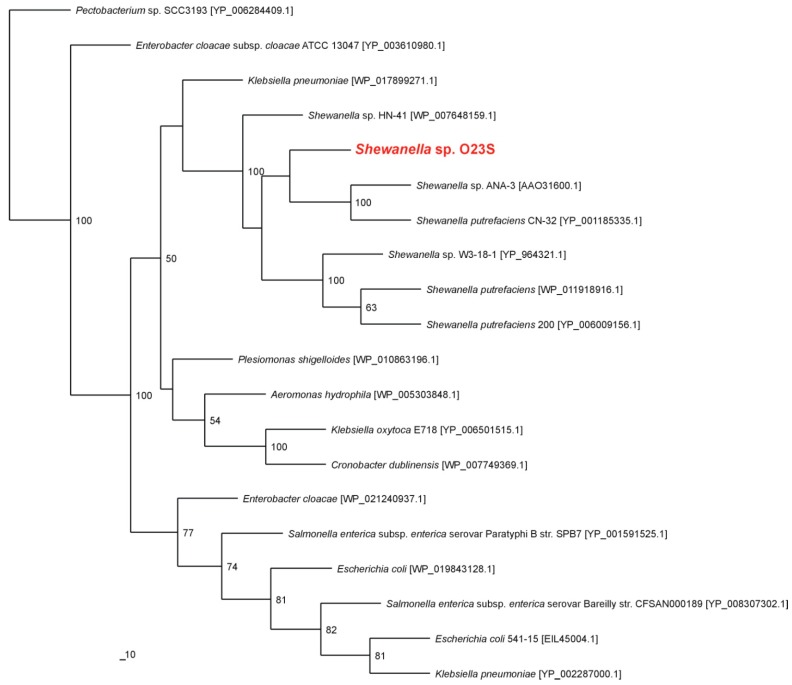
Phylogenetic tree of the cytoplasmic arsenate reductase ArsC based on 20 sequences. The investigated strain is indicated in red. The unrooted tree was constructed using the Maximum Likelihood algorithm according to Jones–Taylor–Thornton (JTT) model. The statistical support for the internal nodes was determined by 100 bootstrap replicates. Accession numbers of the protein sequences used for the phylogenetic analysis are given in parentheses.

The close relationship of the investigated strain with other dissimilatory arsenate reducing *Shewanella* spp. was also confirmed by phylogenetic analysis of cytoplasmic arsenate reductase ArsC. The nearest known phylogenetic relatives of ArsC of *Shewanella* sp. O23S were arsenate reductases of *Shewanella* sp. ANA-3 and *Shewanella putrefaciens* CN-32 (Figure 2).

### 2.2. Growth Analysis and Heavy Metal Resistance

**Figure 3:**
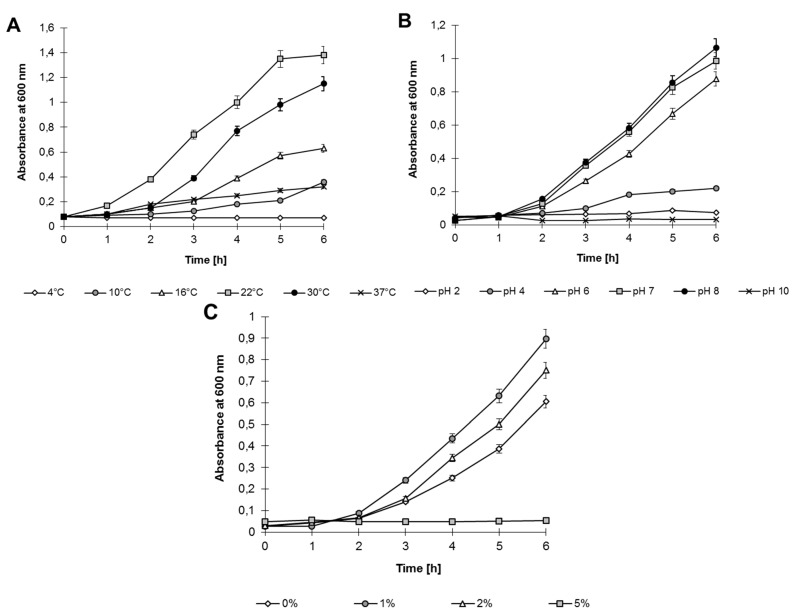
Growth of *Shewanella* sp. O23S under various stress conditions: (**A**) Temperature; (**B**) pH; and (**C**) Salinity.

Analyses performed on Luria–Bertani (LB) medium showed that *Shewanella* sp. O23S can grow under various environmental conditions, in a broad range of: (i) temperatures: 4–37 °C, with the optimum at 22 °C; (ii) pH: 4–8, with the optimum at pH 8; and (iii) salinity: 0%–2%, with the optimum at 1% (Figure 3).

Moreover, Biolog™ analysis revealed that *Shewanella* sp. O23S can utilize a broad spectrum of organic compounds as carbon sources, including pyruvic acid, methyl ester, xylose, galacturonic acid, asparagine, mannitol, 4-hydroxybenzoic acid, serine, glucose, malic acid, putrescine, glycyl-l-glutamic acid, *N*-acetyl-d-glucosamine, l-phenylalanine, and lactose.

**Figure 4:**
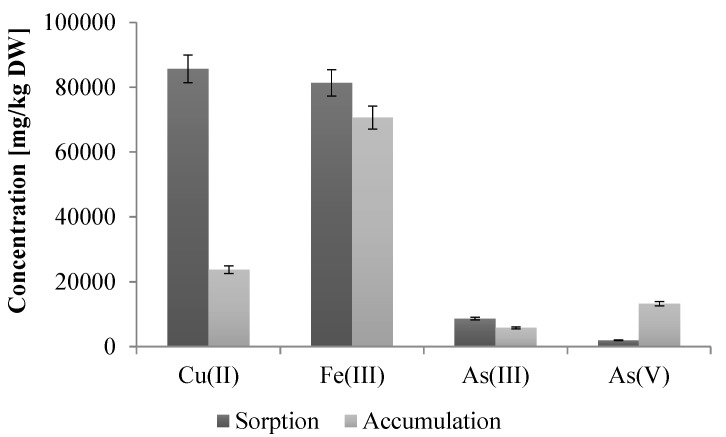
Efficiency of heavy metal sorption (passive process) and accumulation (living biomass) by the cells of *Shewanella* sp. O23S. At the beginning of both experiments the concentration of As(III), As(V), Cu(II), and Fe(III) was 2 mM and the pH of each metal solution was 7.

The preformed analysis also showed that this strain tolerates high concentrations of various heavy metals, including As(III) (up to 10 mM), As(V) (up to 500 mM), Cd(II) (up to 1 mM), Co(II) (up to 2 mM), Cr(III) and Fe(III) (both up to 5 mM), Cu(II) and Zn(II) (both up to 3 mM), Mn(II), Mo(II), Se(VI) and V(V) (all above 20 mM, higher concentrations have not been examined). In turn, the study of the sorption of heavy metals by dead biomass and accumulation by living cells of *Shewanella* sp. O23S showed that only copper and iron are effectively adsorbed (67.4% for Cu(II) and 74.0% for Fe(III)) and accumulated by the cells (18.7% for Cu(II) and 64.2% for Fe(III)) (Figure 4). Lower sorption and accumulation of arsenic species (sorption 5.7% for As(III) and 1.3% for As(V); accumulation 3.8% for As(III) and 8.7% for As(V)) (Figure 4), may indicate that the resistance to these compounds is based on efflux pumps or other active systems rather than the sorption process.

### 2.3. Anaerobic Growth with Sodium Arsenate and Various Electron Donors and Carbon Sources

**Figure 5:**
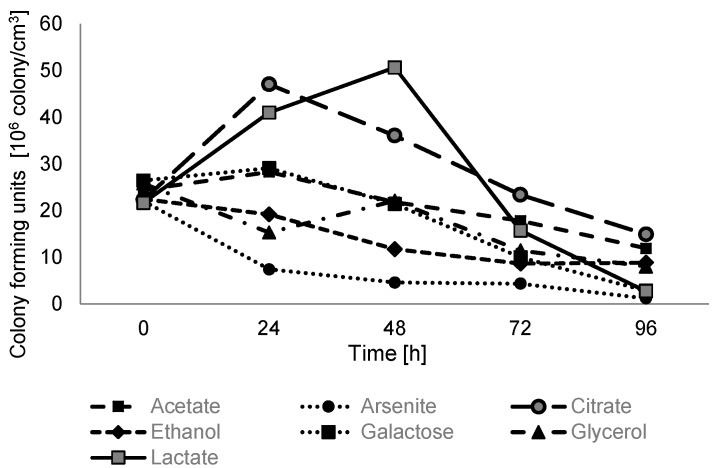
Anaerobic growth of *Shewanella* sp. O23S on minimal salt medium supplemented with sodium arsenate and various electron donors and carbon sources. The strain was grown on minimal salt medium with 2.5 mM sodium arsenate as an electron acceptor and 5 mM of one of the following electron donors: sodium acetate, sodium arsenite, sodium citrate, ethanol, galactose, glycerol, sodium lactate. SD < 5%.

Growth analysis under anaerobic conditions on minimal salt medium supplemented with 2.5 mM of sodium arsenate and 5 mM of various carbon and energy sources showed that sodium lactate and sodium citrate are the only substrates that can be effectively coupled with arsenic respiration (Figure 5).

Complete reduction of 2.5 mM of arsenate coupled with oxidation of 5 mM lactate was observed after 24 h and the growth rate (number of doublings·h^−1^) was calculated to be 0.0349, while the reduction of As(V) with the use of sodium citrate occurred after 48 h and the growth rate was calculated to be 0.0311. In the experiments with other carbon and energy sources, the growth of *Shewanella* sp. O23S and reduction of arsenate was not observed or was very low (galactose, acetate). Based on the above results, all further tests concerning the arsenate reduction process were conducted on lactate.

### 2.4. Kinetics of Dissimilatory Arsenate Reduction

**Figure 6:**
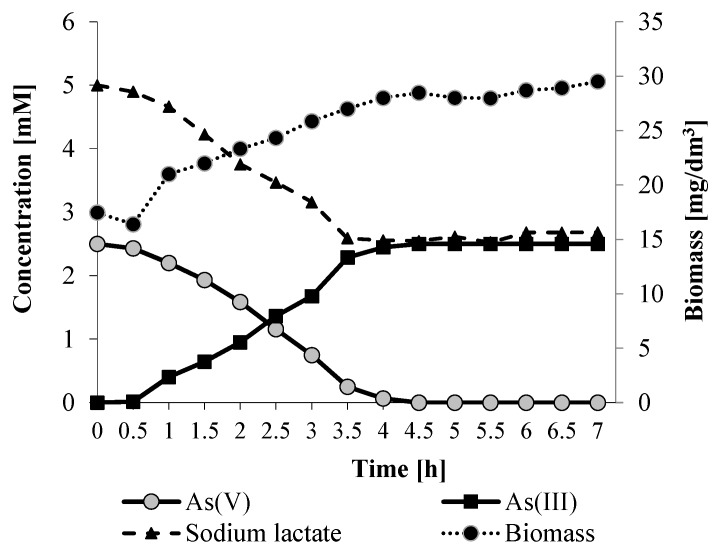
Arsenate reduction mediated by *Shewanella* sp. O23S under anaerobic conditions at 22 °C, pH 8 in minimal salt medium supplemented with 2.5 mM sodium arsenate and 5 mM sodium lactate. SD < 5%.

The above growth experiment revealed that *Shewanella* sp. O23S completely reduced arsenate after one day. Thus, we decided to analyze the kinetics of the As(V) reduction and lactate oxidation within 24 h at optimal conditions (22 °C, pH 8). The performed experiments showed that *Shewanella* sp. O23S grew exponentially for 4 h and then reached the stationary phase. During the exponential phase, complete reduction of 2.5 mM sodium arsenate was observed (Figure 6). The rate of As(V) utilization and the rate of As(III) production were proportional, as bioaccumulation of arsenic by the strain is rather low (Figure 4). Moreover, the reduction of arsenate and the oxidation of sodium lactate was correlated in time (Figure 6), indicating that this process is stoichiometric, and proceeds according to the equation below (1):

(1) $${\text{C}}_{2}{\text{H}}_{4}{\text{OHCOO}}^{-}\;\left(\text{lactate}\right)+{\text{HAsO}}_{4}^{2-}+{\text{H}}_{2}\text{O}\to \;{\text{CH}}_{3}{\text{COO}}^{-}\;\left(\text{acetate}\right)+{\text{HCO}}_{3}^{-}+{\text{HAsO}}_{3}^{2-}+3{\text{H}}^{+}$$

### 2.5. The Effect of Temperature and pH on Dissimilatory Arsenate Reduction

Complete reduction of 2.5 mM of arsenates in liquid minimal salt medium under the optimal conditions was observed after 4 h of culturing. However, laboratory cultures performed on selected substrates do not fully reflect the conditions in the environment, therefore, in order to determine the limits of the adaptability of the strain, a series of experiments focused on defining the physiology of the microorganism were carried out.

In order to determine the effect of temperature on the dissimilatory arsenate reduction, cultures were carried out for seven days at 10, 22, 30 and 37 °C at pH 8. To determine the effect of pH, cultures ware carried out for seven days at 22 °C and the pH was set at 4, 6, and 8. Arsenic concentration, as well as the optical cell density, was tested every 24 h. The results of the selected variants are shown in Table 1 and Table 2.

**Table 1:** Growth of *Shewanella* sp. O23S during dissimilatory reduction of arsenate at pH 8 under various temperature conditions.

Temperature	10 °C	22 °C	30 °C	37 °C
Maximum density of the culture *	0.081	0.190	0.176	0.080
Time required for complete reduction (h)	120	3.5	5.5	nd **
Reduction rate (mg·dm^−3^·h^−1^)	1.562	53.571	34.091	nd **

* Density of the culture was estimated by spectrophotometric measurement at 600 nm; ** Arsenate reduction was not detected.

**Table 2:** Growth of *Shewanella* sp. O23S during dissimilatory reduction of arsenate at 22 °C under various pH conditions.

pH	4	6	8
Maximum density of the culture *	0.109	0.125	0.190
Time required for complete reduction (h)	48	36	3.5
Reduction rate (mg·dm^−3^·h^−1^)	3.906	5.208	53.571

* Density of the culture was estimated by spectrophotometric measurement at 600 nm.

The reduction of As(V) to As(III) was observed at every pH tested, given the temperature was 22 °C. At pH 4, arsenate was slowly reduced, and the reaction was carried out constantly throughout the experiment, for seven days. A definite increase in the reduction rate was observed between the first and the second day of the experiment, correlating with the increase in the growth rate. At pH 6, the reduction was much faster, and was completed after 48 h. The fastest rate of arsenic reduction was observed at pH 8, and As(V) was reduced to As(III) after 24 h or less at 22 and 30 °C. At pH 8, the strain was also capable of growth at 10 °C, and under such conditions, arsenate reduction took 144 h to complete. This result is in accordance with the conditions in the environment from which the microorganism was isolated. In the Gertruda Adit, the pH is in the range of 7.4–8.1, however the temperature in the mine fluctuates between 10 and 12 °C. Although the results of the experiment confirm that the strain can carry out dissimilatory arsenate reduction in the mine, the process is likely to be more efficient at higher temperatures. It is likely that higher temperature causes stimulation of cell growth and increases the intensity of metabolic processes, resulting in a significant acceleration of the arsenate reduction process.

Based on these results, it can be concluded that, for *Shewanella* sp. O23S, the temperature of 22 °C is most favorable for dissimilatory arsenate reduction. At this temperature, arsenate reduction was observed at a pH range of 4–8, and the most intensive growth was observed at 22 °C, at pH 8. The level of pH 8 seems to be optimal for the dissimilatory reduction process.

### 2.6. The Effect of the Presence of Heavy Metals on the Dissimilatory Arsenate Reduction

In order to determine the effect of the presence of heavy metals on the dissimilatory arsenate reduction process, anaerobic cultures on minimal salt medium supplemented with 2.5 mM As(V) (as electron acceptor) and other heavy metals (As(III), Cd(II), Co(II), Cr(III), Cu(II), Mn(II), Mo(II), Se(VI), V(V), and Zn(II)) at concentrations ranging from 1 to 5 mM were carried out. The minimum inhibitory concentration (MIC) was determined as the lowest concentration that completely inhibited growth and arsenic reduction. The results are shown in Table 3.

**Table 3:** Effect of heavy metals on growth and arsenic respiration. Heat map shows the inhibitory effect of heavy metals on anaerobic growth in minimal salt medium supplemented with 2.5 mM sodium arsenate and 5 mM sodium lactate. Each block represents culture density (OD_600 nm_) after 48 h of incubation; + represents complete arsenate reduction (determined by the silver nitrate test).

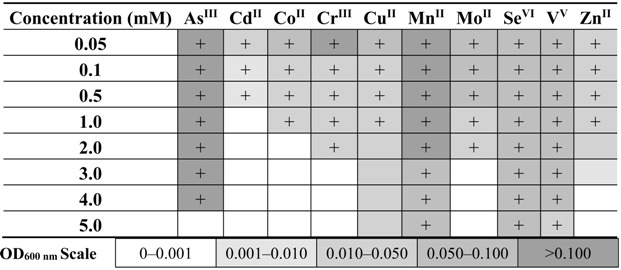

The presence of metals and metalloids slows the rate of arsenate reduction and growth of *Shewanella* sp. O23S, but the rate and scale of the slowdown is strongly dependent on the co-occurring element (Table 3). The greatest impact is made by the presence of Cd, Cu, and Zn, which at relatively low concentrations (0.5–1.0 mM) inhibited the growth and activity of O23S (Table 3). In turn, the addition of 5 mM of Mn(II), Se(VI), V(V), or even As(III) slightly affected or did not affect the arsenate reduction and growth of *Shewanella* sp. O23S. These results correlate with the MIC values previously obtained for the strain grown in the LB medium (see Results 2.2).

### 2.7. Different Terminal Electron Acceptors as an Alternative to Arsenate

**Figure 7:**
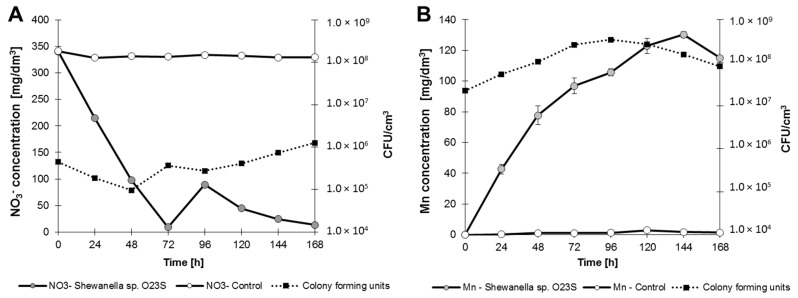

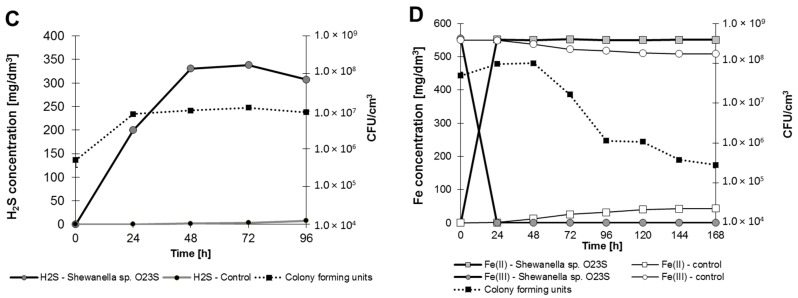
Anaerobic growth of *Shewanella* sp. O23S in minimal salt medium with 5 mM sodium lactate and 2.5 mM of electron acceptors. (**A**) Growth with 5 mM (310 mg NO_3_^−^/dm^3^) sodium nitrate; (**B**) growth with 5 mM (solid phase) manganese(IV) oxide (MnO_2_); (**C**) growth with 5 mM (560 mg S_2_O_3_^2−^/dm^3^) sodium thiosulfate; and (**D**) growth with 10 mM (558 mg Fe/dm^3^) iron(III) citrate. SD < 5%.

The ability to survive and the possibility to effectively compete with the indigenous microflora under anaerobic conditions increases along with the ability to utilize various terminal electron acceptors. Anaerobic growth analysis showed that *Shewanella* sp. O23S (in addition to arsenate) is capable of using nitrate, manganese(IV) oxide thiosulfate, and iron(III) as terminal electron acceptors (Figure 7).

Complete reduction of Fe(III) was observed after 24 h of incubation, while thiosulfate was reduced after 48 h, nitrate after 72 h, and manganese(IV) oxide after 144 h. The highest growth rate was observed when thiosulfate was used as the respiratory substrate (Table 4).

**Table 4:** Growth parameters of *Shewanella* sp. O23S during the dissimilatory reduction of arsenate, iron(III), manganese(IV) oxide, nitrate, and thiosulfate coupled with sodium lactate oxidation.

Terminal Electron Acceptor	Na_3_AsO_4_	MnO_2_	Fe(C_6_H_5_O_7_)	NaNO_3_	Na_2_S_2_O_3_
Number of cells/cm^3^·10^6^ *	95.5	344	100.7	90.05	12.52
Growth Rate (number of doublings·h^−1^)	0.2431	0.0364	0.0227	0.0291	0.1169
Doubling time (h)	2.85	19.04	11.06	23.82	5.93
Time required for complete reduction (h)	4	144	24	72	48

* The maximum number of cells per cm^3^ in the logarithmic growth.

### 2.8. Precipitation of Arsenic Sulfides

The reduction of inorganic compounds in respiratory processes may be beneficial not only for bacteria, but also for the environment. An example of this phenomenon is simultaneous microbial arsenate and sulfate/thiosulfate reduction in waters, which results in precipitation of arsenic sulfides and consequently leads to environmental protection.

**Figure 8:**
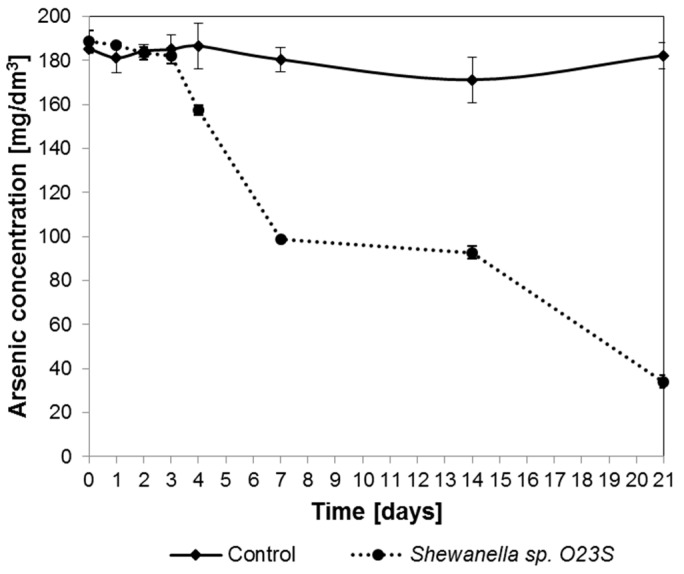
Removal of arsenic from synthetic water containing 5 mM sodium lactate, 2.5 mM (187.5 mg As/dm^3^) sodium arsenate, and 5 mM (560 mg/dm^3^) sodium thiosulfate.

To simulate the environmental conditions, tests were carried out in minimal salt media supplemented with arsenate and lactate, where the following anions were also added: (i) Sulfates and (ii) Thiosulfates. The effect of the presence of these ions on the dissimilatory arsenate reduction process was investigated for 21 days. The results of the experiment were unexpected, as the addition of thiosulfate not only did not inhibit arsenic reduction, but, after the initial three days, led to its precipitation in the reduced form as As_2_S_3_, with a characteristic lemon-yellow color, which was clearly visible after seven days of incubation. Arsenic concentration in the medium (in dissolved form, in supernatant) was tested throughout the experiment and the results are shown in Figure 8. At the end of the experiment, after 21 days, 82.5% loss of arsenic content in supernatant was noted and X-ray diffraction (XRD) analysis confirmed that the observed precipitate is a fine, crystalline arsenic sulfide. The addition of sulfates to the medium did not result in the formation of a precipitate.

## 3. Discussion

In this work, the functional analysis of the *Shewanella* sp. O23S strain, which is capable of dissimilatory arsenate reduction, was performed in the context of the role of DARB in colonization and purification of arsenic-contaminated waters. Based on the 16S rRNA and *arrA* gene amplicons analysis, this strain was found to be dominant in the microbial community in the Zloty Stok mine [7], therefore it was used as a model organism representing DARB. Moreover, phylogenetic analysis of the 16S rRNA gene and ArrA and ArsC proteins revealed a close relationship of the O23S strain with the other known arsenic respiring *Shewanella* spp., making it a good representative of the entire genus.

*Shewanella* sp. O23S, similarly to other DARB [11,12,13], is able to use alternative terminal electron acceptors such as nitrate, thiosulfate, manganese(IV) oxide and iron(III). However, the rate of arsenate reduction is faster than that of other electron acceptors. This result reflects the energetic advantage of arsenate over other compounds, which is also mirrored and noted for other DARB [14]. Such an efficient reduction of arsenate by *Shewanella* sp. O23S may also be associated with the toxicity of arsenic and the need for its removal, since arsenic concentration in Zloty Stok mine waters reaches the level of 5784 µg/dm^3^, which is one of the highest levels measured in surface waters in the world [15].

The *Shewanella* sp. O23S strain investigated in this study was isolated from the microbial community of the Zloty Stok gold mine, which forms complex, multilayer structures, known as microbial mats, with various phylogenetic and physiological groups of bacteria [7]. In microbial communities, such as those in the Zloty Stok mine, the most important from the environment colonization point of view are bacteria, which, in addition to the use of easily assimilable nutrients, have the ability to utilize various toxic organic compounds, radionuclides, and oxyanions, as well as to immobilize heavy metals or metalloids under harsh environmental conditions [16]. *Shewanella* sp. O23S meets these requirements, as it is characterized by a broad spectrum of metabolic activities and is resistant to a variety of stress factors. The performed analyses showed that the strain O23S is capable of growth in a broad range of temperatures, pH, and salinity, and is able to utilize various organic compounds as carbon and energy sources. Colonization of mine waters by *Shewanella* sp. O23S was also possible due to its tolerance of high concentrations of heavy metals, and more importantly, due to the ability of respiratory reduction of various inorganic compounds, with special emphasis on arsenate respiration.

The impact of the O23S strain on the purification of the mine waters must be considered in the context of the activity of the entire microbial community, from which it was isolated. Drewniak [8] showed that the microbial mats found in waters and bottom sediments in the Zloty Stok mine play a significant role in purification of the local environment, as they capture and retain high amounts of arsenic (up to 19,000 mg/kg DW), as well as other heavy metals and pollutants. Among the mechanisms involved in the trapping of heavy metals by microbial mats, the most important are processes independent from metabolic pathways, mainly sorption, but also those related to metabolic activity, leading to the transformation and/or immobilization of heavy metals [17]. In the case of microbial immobilization of arsenic from contaminated waters, there are at least two scenarios by which the process can proceed. In the first, arsenites are oxidized into arsenates, which can be precipitated on iron or aluminum (hydro)oxides and form secondary minerals. The second option assumes that arsenates can be reduced to arsenites, which in turn will be precipitated with sulfides.

By transformation of arsenate into arsenite, *Shewanella* sp. O23S increases toxicity, but allows arsenic to be exported. Moreover, this transformation, in connection with thiosulfate reduction to sulfides, leads to the precipitation of As_2_S_3_, which in turn contributes to the enhancement of arsenic sorption by the microbial mats. It is known that reduction and precipitation processes support the biosorption by microbial mats engaged in water purification from heavy metal ions [18].

**Figure 9:**
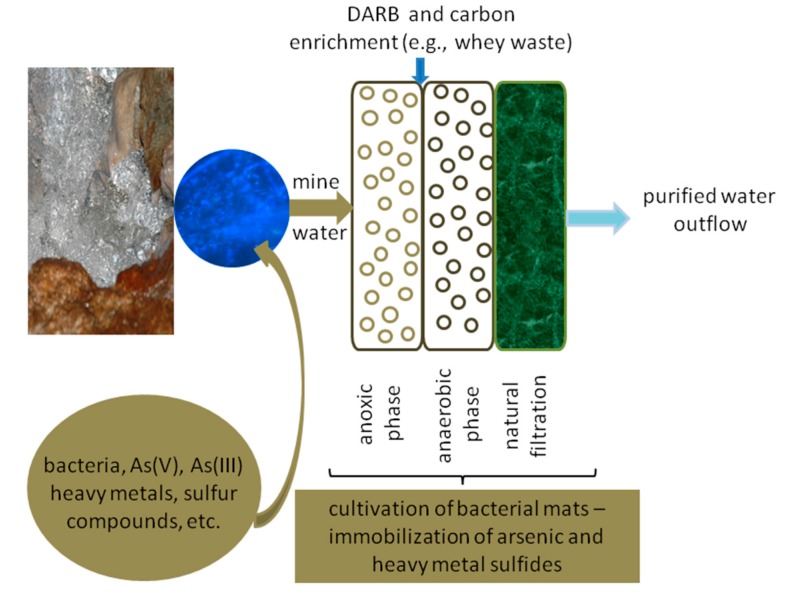
Microbial mats’ culturing system for arsenic removal from waters.

Based on the obtained results, we conclude that *Shewanella* sp. O23S and other DARB strains are the key players of microbial communities responsible for self-cleaning of arsenic-contaminated mine waters. Purification of such waters, as in the majority of other environments, is mainly based on processes related to precipitation, co-precipitation, and sorption, often mediated by microorganisms [19]. The presence of the O23S strain in the environment contributes to the increased precipitation of arsenic and other heavy metals such as sulfides, even without bioaugmentation with sulfate-reducing bacteria. This natural ability may be used for the construction of wetlands, to enhance the purification processes, and increase their efficiency even under conditions unsuitable for many microorganisms. The DARB strains, *i.e*. *Shewanella* sp. O23S, can also be used for the enrichment of naturally occurring microbial mats to form a system capable of arsenic removal from waters (Figure 9).

Culturing or support of growth of *Shewanella* sp. O23S may be carried out in the place of mats’ occurrence and it does not require any special equipment. Therefore, cultivation of such microbial communities is inexpensive, natural, and safe. The functioning of the proposed system is simple. In the first step, all the oxidized forms of arsenic are reduced to arsenite in the presence of thiosulfate or sulfite ions, resulting in their precipitation as sulfides (mainly as As_2_S_3_). The formed precipitate can then be removed by sedimentation or filtration through the sieve-like structure of the mats and is then entrapped by the exopolysaccharide matrix. The purification of water in such systems may be additionally increased by the use of specially prepared peat or bog ores.

## 4. Materials and Methods

### 4.1. Media and Growth Conditions

*Shewanella* sp. O23S was routinely grown in Luria-Bertani (LB) medium [20] or in minimal salt medium [9] (pH 8) supplemented with yeast extract (0.04% *w*/*v*) at 22 °C. Optimal growth parameters were determined in LB medium or minimal salt medium supplemented with sodium arsenate and with appropriate pH (4–8), salinity (0%–2%, adjusted by the addition of NaCl), and heavy metal content (Cd, Co, Cr, Cu, Mn, Mo, Se, V, Zn), and cultured in an appropriate set of temperatures (10–37 °C). For dissimilatory reduction analyses, cultures were propagated in minimal salt medium supplemented with 5 mM sodium lactate and one of the following electron acceptors: 2.5 mM sodium arsenate, 10 mM iron(III) citrate, 5 mM manganese(IV) oxide, 5 mM sodium nitrate, or 5 mM sodium thiosulfate. Analysis of electron donors was performed in the minimal salt medium supplemented with 2.5 mM sodium arsenate and one of the following electron donors: 5 mM sodium acetate, 5 mM sodium lactate, 5 mM sodium citrate, 5 mM galactose, 5 mM glycerol, or 5 mM ethanol. For anaerobic growth, 100 cm^3^ serum bottles with CO_2_:N_2_ (at ratio 20:80) injected into the headspace were used. These bottles were capped with silicon stoppers secured by aluminum crimp seals. Sampling was performed under CO_2_:N_2_ (at ratio 20:80) atmosphere in an anaerobic glove box (Sigma-Aldrich, St. Louis, MO, USA).

### 4.2. Biolog™ Test

The carbon metabolism of the *Shewanella* sp. O23S strain was characterized by the community level physiological profiles (CLPPs) using Biolog™ EcoPlate [21]. Each well of the Biolog™ EcoPlate was inoculated with 100 μL of bacterial suspension and incubated at a constant temperature of 25 °C. The plates were scanned at a wavelength of 600 nm with a Biolog reader (Biolog, Hayward, CA, USA).

### 4.3. Chemical Analysis

Solid samples (bacterial biomass, soil samples) were thoroughly dried at 60 °C and then 9 cm^3^ of 65% HNO_3_ and 1 cm^3^ of 36% H_2_O_2_ were added to a dry weight of 0.25–0.30 g, and digested in a closed system with heating in a microwave oven (Milestone Ethos Plus with Lab Terminal 800 Controller, Sorisole, Italy) [22]. Liquid samples (culture supernatants and liquids) were placed in 12 cm^3^ glass vials and mixed with 65% HNO_3_ at a ratio of 4:1. Quantitative analysis of As, Cu, Fe, and Mn was performed by flame atomic absorption spectrometry (FAAS) and graphite furnace atomic absorption spectrometry (GFAAS) (AA Solaar M6 Spectrometer, TJA Solutions, Cambridge, UK), using a standard solution (Merck, Darmstadt, Germany) prepared in 0.5 M HNO_3_. Arsenic species in culture supernatants were determined as described by Drewniak, *et al.* [23].

### 4.4. High Performance Liquid Chromatography (HPLC)

Arsenic speciation and lactate concentration were analyzed by high-performance liquid chromatography (Waters, Milford, MA, USA) on a reversed-phase polymeric resin (Waters IC-PakTM Anion HC column (150 by 4.6 mm; pore size, 10 μm)) with a 25 mM phosphate buffer as the mobile phase and a flow rate of 0.8 cm^3^/min. Arsenic speciation was determined using Waters Photo Diode Array Detector (W2998) at 193 nm. Standard curves for the analysis of samples were prepared based on the minimal salt medium containing a known concentration of one of the arsenic species or lactate. The results were analyzed using the Empower software (Waters).

### 4.5. Qualitative Assay of Arsenic Speciation (Silver Nitrate Test)

The reaction between AgNO_3_ and As(III) and As(V) results in the formation of a colored precipitate. A brown precipitate indicates the presence of Ag_3_AsO_4_ (silver ortoarsenate), while a lemon-yellow precipitate indicates the presence of Ag_3_AsO_3_ (silver arsenite). The culture supernatant (500 cm^3^) was collected and mixed at a ratio of 1:1 with a 0.1 M silver nitrate(V) solution. The color of the precipitate indicated which form of arsenic is present in the sample.

### 4.6. Determination of NO_3_^−^ Concentration

The concentration of nitrate ions in the solution was determined using a Nitrate/Nitrite 4 colorimetric kit (Nanocolor, Machery-Nagel, Düren, Germany). The method is based on a color reaction between the nitrate ions and sulfanilamide and *N*-(1-naphthyl) ethylenediamine (freeze-dried), giving rise to a red-violet azo dye. Concentration measurements were performed on a Pf-12 spectrophotometer (Machery-Nagel) at a wavelength of 540 nm.

### 4.7. Detection of Hydrogen Sulfide

For the detection of hydrogen sulfide and a rough estimation of its concentration, a DP-24 meter (Nanosens, Tarnowo Podgorze, Poland) was used. Measurements were performed as recommended by the manufacturer.

### 4.8. Determination of Fe^2+^ Concentration

To determine the concentration of ferrous ions in the solution, a colorimetric method was used. The method relies on the specific reaction of ferrous ions with colorless *o*-phenanthroline, resulting in an orange-red chelate complex. The reaction was performed in an acetate buffer, stabilizing the formed complex (pH in the range of 2–9). A standard curve was prepared based on the analysis of the minimal salt medium samples containing a known concentration of Fe^2+^ ions. The detection range of this colorimetric method for cations is in the range of 0.001–0.1 mg/dm^3^, therefore the samples were appropriately diluted before measurements. The absorbance was measured at a wavelength of 510 nm using a Shimadzu 1800 Spectrophotometer (Shimadzu Corporation, Osaka, Japan).

### 4.9. Determination of the Total Fe Concentration

To determine the total concentration of iron ions in the solution, the colorimetric method was used (Nanocolor kits). The method is based on a two-step reaction. Firstly, all ferric ions are reduced to ferrous ions. The second step is the specific reaction of ferrous ions with triazine, yielding a violet complex. Concentration measurements were performed on an FP-12 spectrophotometer at a wavelength of 540 nm. This method has been validated by comparing the colorimetric assay results with those obtained from AAS measurements.

### 4.10. Determination of SO_4_^2−^ Concentration

The sulfate concentration was determined by the colorimetric method using a Spectroquant kit (Merck). This method is based on the color reaction, resulting in the formation of a brown-red complex. Spectrophotometric concentration measurements were performed at a wavelength of 515 nm using a Shimadzu 1800 Spectrophotometer.

### 4.11. XRD Analyses

A Philips X’Pert PW 3020 X-ray diffraction analyzer (Philips, Almelo, The Netherlands) was used to characterize the mineral composition of precipitate from a 21-day anaerobic culture with lactate and thiosulfate. Cu KR radiation (1.54056 Å) was used at 40 kV and 45 mA.

### 4.12. Determination of the Minimum Inhibitory Concentrations of Metals/Metalloids

To examine the minimal inhibitory concentrations (MIC) of heavy metals, 96 well microplates containing LB medium amended with the respective heavy metal compounds were used. Each well of a microplate was inoculated with cells from fresh overnight cultures to a final density of approximately 10^6^ cells/cm^3^ (OD_600 nm_ = 0.06) and then incubated for 48 h at 22 °C when OD_600 nm_ was checked again. The following metals and their compounds were used for MIC determination: Na_2_HAsO_4_ 0.0–20 mM; NaAsO_2_ 0.0–500 mM; CdSO_4_·8H_2_O 0.0–5.0 mM; CoSO_4_·7H_2_O 0.0–5.0 mM; Cr_2_(SO_4_)_3_·18H_2_O 0.0–20 mM; CuSO_4_·5H_2_O 0.0–10.0 mM; FeCl_3_ 0.0–5.0 mM; MnSO_4_·H_2_O 0.0–20 mM; MoSO_4_ 0.0–20 mM; Na_2_SeO_4_·10H_2_O 0.0–20 mM; NaVO_3_ 0.0–20 mM; ZnSO_4_·7H_2_O 0.0–5.0 mM. The MIC was defined as the lowest concentration of Me*^n^*^+^ that completely inhibited bacterial growth.

### 4.13. The Influence of the Presence of Heavy Metals on Arsenate Reduction by the Shewanella sp. O23S Strain

The experiment was performed in 96 well plates using a modified minimal salt medium, supplemented with sodium lactate (5 mM), yeast extract (0.004%), Touvinen salts (0.2%), 2.5 mM As(V), and with salts of the following heavy metals: As(III) 0.0–4 mM; Cd(II) 0.0–5 mM; Co(II) 0.0–5 mM; Cr(III) 0.0–5 mM; Cu(II) 0.0–4 mM; Mn(II) 0.0–5 mM; Mo(II) 0.0–20 mM; Se(VI) 0.0–20 mM; V(V) 0.0–5 mM; and Zn(II) 0.0–3 mM. Inoculation was carried out analogously to the MIC test. The cultures were grown under anaerobic conditions for 48 h at 22 °C and the changes in the number of cells was monitored by OD_600 nm_ measurements. The reduction of arsenate was verified with the silver nitrate test. The increase in optical density coupled with arsenate reduction was considered as a positive result.

### 4.14. Sorption and Accumulation Experiments

To determine sorption abilities, the strain O23S was inoculated on 100 cm^3^ liquid Luria-Bertani medium, and cultured for 24 h at 22 °C. Then, 50 cm^3^ of the culture was centrifuged (5 min, 10,000× *g*), dried (24 h/60 °C) to constant weight, and the obtained pellets were re-suspended in 20 cm^3^ of 0.9% NaCl containing As(III), As(V), Cu(II), or Fe(III) at the final concentration of 2 mM and pH 7. After 2 h of incubation at 22 °C, pellets were centrifuged (5 min, 10,000× *g*), dried (24 h/60 °C), and mineralized (24 h/60 °C) by the addition of 1 cm^3^ of 69% HNO_3_ to the sample. Subsequently, the supernatants and mineralized pellets were analyzed by AAS.

To determine accumulation abilities, the strain O23S was cultured for 72 h at 22 °C in 200 cm^3^ of LB medium supplemented with 2 mM solution of the respective heavy metal salts (sodium arsenite, sodium arsenate, copper sulfate, and ferric chloride). After 72 h of incubation, cells were harvested by centrifugation (5 min, 10,000× *g*), dried (24 h/60 °C) to constant weight, and the pellets were mineralized, centrifuged (5 min, 10,000× *g*), and examined using GFAAS. Supernatants were analyzed using FAAS.

### 4.15. Test for Arsenic Removal by Arsenic Sulfide Precipitation

The cultures were carried out in special bottles capped with rubber septa under anaerobic conditions obtained by washing the medium with a CO_2_:N_2_ mixture (at a ratio of 20:80). Experiments were performed in minimal salt medium (pH 8), supplemented with sodium lactate (5 mM), yeast extract (0.004%), Touvinen salts (0.2%), 2.5 mM As(V), and 5 mM sodium thiosulfate, or the same amount of sodium sulfate. Inoculation was carried out by adding approximately 10^6^ bacterial cells per cm^3^ to the medium. The experiment was carried out for 21 days. The formation of a yellow precipitate was observed and the amount of arsenic present in the supernatant was determined by GFAAS.

### 4.16. DNA Manipulations, PCR Amplification, and Sequencing

Standard DNA manipulation methods were performed as described by Sambrook and Russel [20]. Amplification of the 16S rRNA genes and the *arsC* genes was performed as described by Drewniak, *et* *al.* [24]. For the amplification of the *arrA* genes, primer pair ArrAfwd and ArrArev was used as described by Malasarn, *et al.* [25]. The PCR products were ligated with the vector pGEM-T-Easy (Promega) and were transformed to chemically competent *Escherichia coli* TG1 cells. Plasmid inserts were sequenced on an ABI3730 DNA analyzer (Applied Biosystems, Foster City, CA, USA) at the Laboratory of DNA Sequencing and Oligonucleotide Synthesis, IBB PAS, using universal M13F and M13R primers.

### 4.17. Phylogenetic Analysis of 16S rRNA Gene, arrA and arsC Genes

The near full-length 16S rRNA gene sequences (~1.4 kbp) from the isolates and reference sequences from the known arsenic-utilizing bacteria were aligned using ClustalW software (www.ebi.ac.uk/clustalw). The obtained alignment was adjusted manually and then used to construct a phylogenetic tree. The unrooted tree was constructed using the distance matrix Neighbor-Joining Method with NEIGHBOR software from the PHYLIP 3.6 package [26]. Distance matrices were calculated by DNADIST (PHYLIP) using the Jukes-Cantor formula. Bootstrap analysis was carried out 1000 times using SEQBOOT (PHYLIP). A consensus tree was computed with CONSENS (PHYLIP).

## 5. Conclusions

The *Shewanella* sp. O23S strain characterized in this study constitutes a viable element of the microbial community of the Zloty Stok gold mine, and the driving agent of the self-purification of waters contaminated with arsenic. Due to its phylogenetic proximity to other DARB, it is a good model organism for studying the role of dissimilatory arsenate reduction in the biogeochemical cycle of this element, especially in the context of its removal from the environment. The occurrence of the strain in the microbial mats increases the efficiency of water self-purification, and therefore, it can be used for the enrichment of other bioremediation sites, such as wetlands. Microorganisms used for the construction of the proposed culturing system for arsenic removal from waters should utilize simple organic nutrients, as well as various toxic organic compounds, radionuclides, and oxyanions. They should also immobilize heavy metals or metalloids under extreme environmental conditions. *Shewanella* sp. O23S meets all these requirements, as it is characterized by a broad spectrum of metabolic activities and is resistant to a variety of stress factors.
